# INES: Interactive tool for construction and extrapolation of partitioned survival models

**DOI:** 10.1186/s12962-023-00456-6

**Published:** 2023-07-31

**Authors:** Vicente Gimeno-Ballester, Daniel Perez-Troncoso, Antonio Olry-Labry, David Epstein

**Affiliations:** 1 Servicio de Farmacia, Hospital Universitario Miguel Servet, Zaragoza, Spain; 2 Agència De Qualitat I Avaluació Sanitàries De Catalunya, Barcelona, Spain; 3 Escuela Andaluza de Salud Pública, Granada, Spain; 4 Centro de Investigación Biomédica en Red de Epidemiología y Salud Pública, Granada, Spain; 5 Ministry of Health, Madrid, Spain; 6 Department of Applied Economics, University of Granada, 18071 Granada, Spain

**Keywords:** Partitioned survival model, Cost-effectiveness analysis, Economic evaluation, Extrapolation, Survival analysis

## Abstract

**Background:**

INES (INteractive model for Extrapolation of Survival and cost) provides an open-access tool powered by R that implements three-state partitioned survival models (PSM). This article describes the properties of the tool, and the situations where INES may or may not be suitable.

**Methods:**

INES is designed to be used by investigators or healthcare professionals who have a good grasp of the principles of economic evaluation and understand the strengths and weaknesses of partitioned survival models, but are not sufficiently familiar with a statistical package such as Excel or R to be able to construct and test a de-novo PSM themselves.

INES is delivered to the user via a batch file. Once downloaded to the user’s hard drive, it interacts with the user via a portable version of R with web interactivity built in Shiny. INES requires absolutely no knowledge of R and the user does not need to have R or any of its dependences installed. Hence the user will deal with a standalone Shiny app. Inputs (digitalized survival curves, unit costs, posology, hazard ratios, discount rate) can be uploaded from a template spreadsheet.

**Results:**

The INES application provides a seamlessly integrated package for estimating a set of parametric hazard functions for progression free and overall survival, selecting an appropriate function from this menu, and applying this as an input to a PSM to calculate mean costs and quality-adjusted life years. Examples are given that may serve as a tutorial.

**Conclusion:**

INES offers a rapid, flexible, robust and transparent tool for parametric survival analysis and calculating a PSM that can be used in many different contexts.

**Supplementary Information:**

The online version contains supplementary material available at 10.1186/s12962-023-00456-6.

## Background

Models are decision tools commonly used for the economic evaluation of health care technologies. In general terms, the model framework might be configured in several ways including decision tree, partitioned survival model (PSM), state-transition (Markov cohort) model, or event -based simulation [1]. This article presents a new open-access modelling tool for PSM: INES (INteractive model for Extrapolation of Survival and cost).

The process of constructing a PSM usually requires three fundamental steps. First, the individual patient data (IPD) must be available from a clinical study containing duration of overall survival (OS) and progression-free survival (PFS) in the intervention and control group. However, IPD are often not available to the researcher. Hence the first step in these situations consists of recreating the IPD from the coordinates of published OS and PFS Kaplan–Meier curves using a statistical algorithm, a process known as “digitalization” of the survival curves. The second step is to estimate a set of parametric functions fitted to the IPD, and for the user to select the most appropriate function (we discuss possible criteria in the Methods). The third step is to estimate mean costs and mean (quality-adjusted) life years over a chosen time horizon. In a PSM this is achieved extrapolating the survival functions over the model time horizon, dividing the analysis time into short model cycles, applying cost weights, utility weights and a discount factor to the proportion of the initial cohort in each state in each cycle, and numerically integrating over the model time horizon to obtain the areas under the curves.

There are several existing packages available to conduct the final step in a PSM analysis, such as TreeAge®, R packages [2], or the user could construct a *de-novo* model in Excel or R. These options vary in the degree of technical and programming expertise required of the user. However, all of these software options start from the premise that the user will provide the package with the parametric survival functions as inputs. In other words, existing PSM packages take for granted that the user has successfully accomplished the first two steps. This poses a steep learning curve and may be a barrier to many practitioners.

INES is designed to be used by investigators who have a good grasp of the principles of economic evaluation and understand the strengths and weaknesses of partitioned survival models, but do not have the time, statistical background or programming expertise to conduct all these steps for a PSM themselves. It offers a rapid, flexible, robust tool that brings together these modules in a single, seamless package. This article describes the properties of the tool, and discusses the situations where it may or may not be suitable. This article illustrates the tool using a particular case for demonstration purposes but we do not make any statement here about the appropriateness of these specific modelling choices or the cost-effectiveness of particular therapies.

## Methods

INES is delivered to the user via a batch file, available from https://freeinesapp.github.io [3]. Once downloaded to the user’s hard drive, it interacts with the user via a portable version of R (v. 4.1.3), with web interactivity built in Shiny [4]. INES requires no knowledge of R and the user does not need to have R or any of its dependencies installed (in practice, the user will deal with a standalone Shiny app). INES can be run on Windows (7, 8, 10 and 11), Mac and Linux. The tool is available free of charge and under a creative commons attribution license (CC BY).

Once downloaded and extracted to a folder on the user’s hard drive, INES is initiated by selecting “run.bat” from the folder. Data are input via a spreadsheet template provided in the batch file. Once data are inputted, run time is a few seconds.

**Fig. 1:**
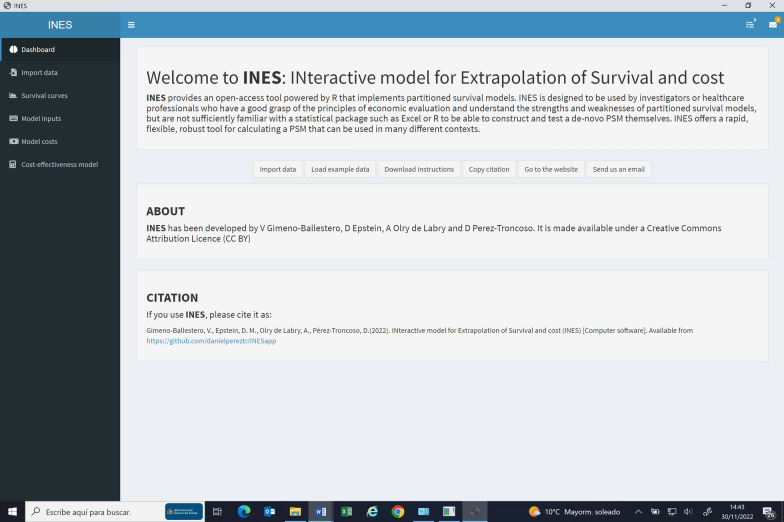
Dashboard

INES is designed to accomplish the tasks summarized in Table 1 and described in more detail in the following sections. The PSM as enacted in INES estimates the proportion of the cohort in each of 3 states (labelled progression-free, progressed and dead) using two survival curves (PFS and OS), where the difference represents the time post-progression (PPS). The terms progression-free and progressed are for convenience: fundamentally a PSM assumes that backward transitions are not allowed. Patients receive the intervention of interest at the start of the model or during the PFS state and may employ another treatment during the next state. Quality of life (utility) and cost weights are associated with the time in the progression-free and post-progression states [5]. INES compares two alternative therapies. In this article they will be termed “intervention” and “comparator” or “control”. Users are guided through the steps required by an interactive dashboard (Fig. 1). The model cycle length in INES is one month, and this cannot be altered. Data can be uploaded via a spreadsheet template, described in Table 2. Table 3 justifies the modelling choices built into INES.

**Table 1:** Functionality of the INES tool

Function	Description	Reference to method or source of R code
Derive the IPD	Derives from the published Kaplan Meier survival curves a close approximation to the original individual patient time-to-event data (IPD) for OS and PFS for 2 treatment groups	IPDfromKM
Estimate parametric survival curve in control group	Allow the user to choose from a menu of parametric survival functions fitted to the IPD for PFS and OS in the control group	flexsurvreg
Model fit: AIC	Calculate the AIC for the fit of the data to the parametric model in the control group (PFS and OS)	flexsurvreg
HR	Calculate the hazard ratios between treatment groups, assuming proportional hazards (PFS and OS)	coxph
HR	Perform a test of proportional hazards (PFS and OS)	cox.zph
Parametric survival curve in intervention group	Estimate the parametric survival curves in the intervention group by applying the hazard ratio to the respective control group survival curve (PFS and OS). The user is given the option either to use published HR or the HR calculated by the package	Heemod:: apply_hr
Model fit: Visual	Allow the user to visually inspect each parametric survival curve extrapolated over the chosen time horizon, and compare to the observed data (Kaplan Meier survival curve) (PFS and OS in each treatment group)	ggplot2
Costs	Allow the user to specify the unit costs of medicines and other resources and the number of units employed per day, week or month, in each treatment group during the progression-free and post-progression states	heemod:: create_parameters_ from_tabular
Utility	Allow the user to specify the utility weights in each treatment group during the progression-free state, and a single utility weight during post-progression. The model will calculate life years if the utility weights are set to 1	heemod:: create_parameters_ from_tabular
Discount rate	Apply chosen discount rate to costs and effects (equally)	Heemod:: discount
PSM	Calculate the PSM in each treatment group over the specified time horizon	Heemod::run_model
Sensitivity analysis	Allow user to specify the parameters for univariate sensitivity analysis within a range	Heemod::run_dsa
Results: graphical	Allow user to visualise and download graphs of state membership (PFS, PPS, death) over time in each treatment group	plot, panels = by_state
Results: table	Show undiscounted and discounted mean costs and effects in each treatment group and the incremental cost-effectiveness ratio	
Sensitivity analysis results: graphical	Allow user to visualise and download a tornado chart of the sensitivity analysis	ggplot2

*IPD* individual patient data. *OS* overall survival. *PFS* progression free survival. *PPS* Post progression survival. *AIC* Akaike Information Criterion. *HR* hazard ratio. *PSM* partitioned survival model

**Table 2:** Data required by the INES tool

Parameter	Description	Usual source	Univariate sensitivity analysis
Probabilities of survival in PFS and OS in intervention and control	Spreadsheet table of probabilities at monthly time points	Numerical data from published Kaplan–Meier curves. These can be extracted using the WebPlotDigitizer tool	No
Number at risk	Spreadsheet table of number at risk during the follow up of the clinical study	Usually available alongside published Kaplan–Meier curves	No
Number of events (optional)	The number of events for each treatment group and outcome (deaths, and progression-or-death). This can be left null	Usually available alongside published Kaplan–Meier curves. This data can increase the precision of the simulation of the IPD, but is not essential	No
Hazard ratio	The hazard ratio for PFS and OS comparing the intervention with the control	Clinical trial reports or literature. The user has the option of either using published values obtained from a clinical trial report or meta-analysis, or calculating the HR based on the data provided to the model	Yes
Discount rate	A number, usually between 0 and 6%. It must be expressed as the annual rate. The INES tool will automatically convert to monthly equivalent	Official guidelines for benefit–cost analysis in each country	Yes
Unit costs	The cost or price per unit of each resource included in the costs	Official sources or literature	Yes
Resource use during the PFS state	The number of units consumed for each of the resources included in the costs, in each treatment group, during PFS. Consumption can vary over time. The user can express the consumption in units per day, week or month	Clinical trial reports or literature	No
Resource use during the post progression state	The number of units consumed for each of the resources after progression (by initial treatment group). Consumption cannot vary over time. The user can express the consumption in units per day, week or month	Clinical trial reports or literature	No
Utility weights during the PFS state	Utility weights in each treatment group during the progression-free state, Weights cannot vary over time. The model will calculate life years if all the utility weights are set to 1	Clinical trial reports or literature	Yes
Utility weights during the post progression state	Utility weight during post-progression. Weights cannot vary over time or vary by initial treatment. The model will calculate life years if all the utility weights are set to 1	Clinical trial reports or literature	Yes

**Table 3:** Model choices in INES and possible alternative configurations of a PSM

Configuration in INES	Possible alternative modelling choices	Reason for not employing the alternative configuration in INES
The hazard function in the intervention is the same as the control	Allow independent hazard functions in each treatment group	Complexity: Would require the user to choose 4 parametric functions rather than 2 Validation: Predictions made by the model cannot be explained with reference to published HR
Proportional hazards are assumed	Allow the HR to change over time	Complexity: Would require the user to specify a function for how the HR changes over time (continuously or piecewise) during and after the clinical study. These parameters are rarely estimated in clinical studies
INES provides the option of estimating the hazard ratios from the observed data. These HR are estimated using the Cox model	Estimate the hazard ratios using the corresponding parametric model (flexsurvreg) rather than the Cox model	Validation: The HR would be different for each parametric model, and might be substantially different to the value published in a clinical study, which is usually also estimated by a Cox model
The utility weights are constant over time within treatment groups	Allow utility weights to vary over time, for example, to take account of adverse treatment-related events during chemotherapy	Work in progress: This may be considered for a future release of the model
No probabilistic sensitivity analysis	Allow probabilistic sensitivity analysis, including model averaging of different options for the survival function	Work in progress: This may be considered for a future release of the model

### Digitization of the Kaplan–Meier survival curves

INES requires as input the numerical coordinates of the Kaplan–Meier survival curves (the probability of OS and PFS state membership at each month). These can be digitized from publications using software such as WebPlotDigitizer (the website provides manuals and video tutorials [6]). These coordinates must be accompanied by the numbers at risk and, if available, the aggregate number of events. Behind the scenes, the INES tool uses a published R algorithm to map the digitized curves back to the original individual patient data (IPD), the time at which events occurred or censoring occurred [7, 8]. INES in its current configuration does not accept the original IPD as input.

### Estimation of parametric survival functions in the control group

A PSM model requires as an input a parametric survival function for PFS and OS. Parametric functions are recommended because published PFS or OS survival curves in clinical studies are rarely complete. For example, many therapies gain marketing authorization before even half of the patients in the study have died. Notwithstanding, economic evaluation requires an estimation of mean survival time in each state. Even if available, median survival time will not be a suitable proxy for mean survival time if some individuals have long survival times after the median [9]. Hence it is necessary to model and extrapolate the PFS and OS survival curves.

Different parametric functions can make very different predictions about survival, especially in the time after the clinical trial. This is a crucial area of uncertainty in the construction of any PSM. There are several options for how survival might be modelled. Guidelines recommend that the chosen function should approximate both to the observed clinical study data (internal validity), and the investigators’ understanding of the long-term progression or survival of the target population of patients (external validity) [10].

INES is unique among economic evaluation tools as it provides an integrated package that enables the user to estimate a set of parametric hazard functions from observed PFS and OS Kaplan–Meier coordinates, select an appropriate function from this set, and apply this function as an input to a PSM to calculate mean costs and quality-adjusted life years. All the algorithms are handled behind the scenes and the user is not required to manipulate any R commands.

There are 4 survival functions to consider: OS and PFS in each treatment group. In general terms, one could fit independent functional forms to each of the 4 curves, or the same functional form, or permutations thereof [11]. The particular approach taken in INES is to fit parametric survival functions to model survival in the control group, and apply a hazard ratio (HR) to represent the respective survival in the intervention group (Table 3). The INES tool allows the user to choose from one of eight functions: exponential, gamma, log-logistic, Weibull, log-normal, Gompertz, generalized gamma, and generalized-F. Different parametric functions can be chosen for PFS and OS.

**Fig. 2:**
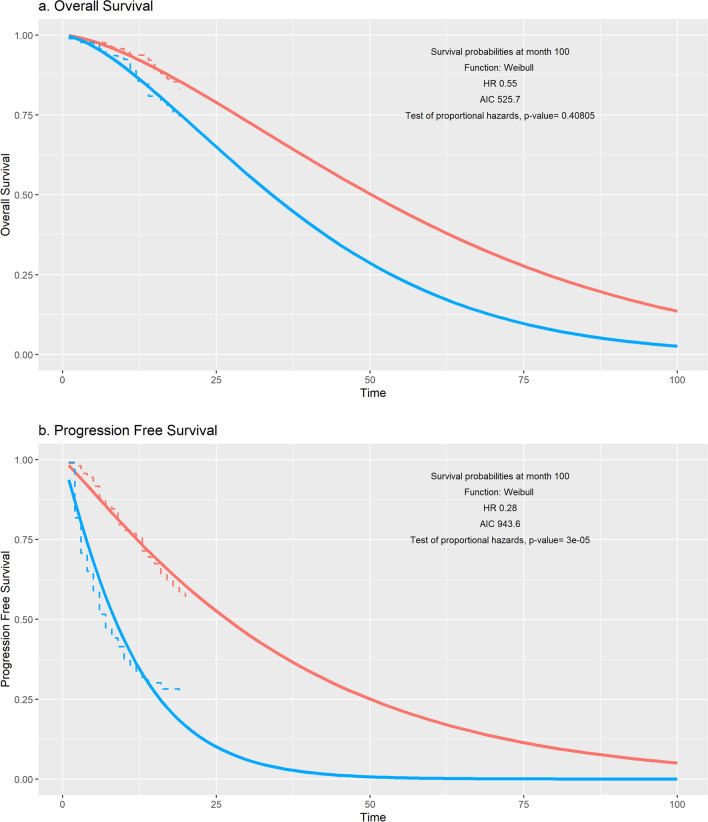
**a** Overall survival and **b** Progression-Free Survival predicted by the INES tool. Red curve: intervention group. Blue curve: control group. Continuous curve: predicted survival. Intermittent curve: observed survival (Kaplan Meier). *AIC* Akaike Information Criteria. *OS* overall survival. *PFS* Progression Free Survival

The INES tool offers two useful features to help the user choose an appropriate parametric function. First, INES calculates the Akaike Information Criteria (AIC) (Fig. 2). This compares the fit of the observed comparator group data with the predictions of the parametric model. The AIC rewards goodness of fit of the parametric function to the observed data, while penalizing the number of parameters. However, AIC (and similar tests) only measure internal validity, and should be used cautiously. Such tests tell us nothing about the absolute quality of a model, only the relative quality of the candidate parametric functions. The AIC does not provide any warning that all models might fit the data poorly. The AIC also tells us nothing about the ability of the model to predict events that occur after the source data follow-up. The second useful feature provided by INES is a graph of the observed data and the predicted model. This allows the user to visualize the fit of the parametric function with the observed data, and to examine the effect of extrapolating this function over the model time horizon (Fig. 2). The user should seek expert judgement about the plausibility of the chosen model.

### Hazard ratio for PFS and OS

The INES tool assumes the hazard ratios for OS and PFS are unchanged over time (proportional hazards, PH). The user can use the spreadsheet template to input hazard ratios for PFS and OS into INES based on published evidence from a clinical study or the literature. Alternatively, the user can request that INES calculates the hazard ratios from the IPD (Cox PH model).

The INES tool uses the apply_hr function from the package HEEMOD to calculate the survival function in the intervention group, given the survival function in the control group and the HR. The apply_hr function multiplies the hazards in the control group by the (constant) HR. This works whether the control group model was estimated by flexsurv using the PH metric (that is, exponential or Gompertz) or the accelerated failure time metric (other models).

Proportional hazards mean that the rate of events (the hazards) in individuals in the intervention arm are a constant multiple of the rate in the control arm. As noted above, the PH assumption is only one of the possible options for modelling survival curves in a PSM and extrapolating beyond the clinical study [10–12]. The user must consider whether the PH assumption is appropriate. A visual inspection of the Kaplan–Meier survival curves in each group can be informative. If the survival curves cross, or if the survival curves radically diverge, then this may indicate that hazards may not be proportional. The INES tool also provides a statistical test based on the digitized survival curves [13] (Fig. 2). If the p-value is low (e.g. less than 0.05) then the sample data are not consistent with the null hypothesis of proportional hazards. However, statistical tests are not the only criteria and users must seek expert judgement to assess whether the proportional hazards model is appropriate for modelling and extrapolation in their evaluation. If hazard ratios are not thought to be constant over the model time horizon, then the user should consider using a different tool.

### Discount rate

The discount rate ensures that the model takes account of social time preference [14]. The tool uses the same discount rate for costs and benefits, and the value should be input as an annual rate (guidelines usually recommend values between 0 and 0.05 [15]). A range can be employed for univariate sensitivity analysis.

### Unit costs

The tool requires users to introduce prices or unit costs of each of the resources employed (see "Resource use" section). A range of values can be employed for univariate sensitivity analysis.

### Resource use

The tool allows users to introduce the number of units of resources employed in each treatment group in each health state. These can be doses of medicines, adverse events, diagnostic tests, other healthcare or any other type of resource. The user can express the consumption in any period either at a general level (e.g. 1 unit per month) or at a very granular level (e.g. on specific days). The INES tool will automatically and precisely translate this information into the equivalent consumption during each model cycle.

During the PFS state, INES allows consumption of each resource to vary over time. For example, in the ANDROMEDA trial [16], patients received daratumumab in “treatment cycles” (not to be confused with the “model cycle” which is an entirely different concept). Each treatment cycle in ANDROMEDA lasted 4 weeks. Patients received daratumumab once a week in treatment cycles 1 and 2, every 2 weeks in treatment cycles 3 through 6, and every 4 weeks thereafter until disease progression, the start of subsequent therapy, or for a maximum of 24 treatment cycles from the start of the trial, whichever occurred first. In the clinical study, other therapies were also given in both treatment groups, but are not discussed here for brevity. INES has been designed to be flexible enough to calculate consumption under complex administration regimens such as these. Additional file 1 shows the data input template for this example and gives further explanation of the variables.

### Utility weights

The model requires the user to enter three utility parameters: the utility weights in each treatment group during the progression-free state, and a single utility weight during post-progression. The model will calculate life years if the utility weights are 1. A range can be employed for univariate sensitivity analysis. The current version of the tool does not allow utility weights to vary over time, but this might be modified in future versions.

### PSM model engine

The model engine for the PSM is implemented by the HEEMOD package in R (Table 1) [17]. The user provides the chosen parametric survival functions for PFS and OS ("Estimation of parametric survival functions in the control group" section), the hazard ratios ("Hazard ratio for PFS and OS" section), the discount rate ("Discount rate" section), resource use quantities and prices of those quantities ("Unit costs" and "Resource use" section) and the utilities ("Utility weights" section). Behind the scenes, HEEMOD then defines the cost and utility weights for the three states (PFS, PPS and dead) (define_state), the strategies (define_strategy) and runs the model (run_model). The probability of membership of the PPS state as the difference between OS and PFS is set up using the define_part_surv function. A half-cycle correction is applied, meaning that events are calculated as if they occur half-way through each month. Univariate sensitivity analyses are conducted using the run_dsa function.

### Probabilistic sensitivity analysis (PSA)

The current version of the tool does not implement PSA. While HEEMOD provides a function to implement PSA based on the same variables used in the univariate sensitivity analysis (Table 2: unit costs, hazard ratios, and the discount rate), it does not account in PSA for what is often the principal source of uncertainty, namely the choice of parametric survival function ("Estimation of parametric survival functions in the control group" section). Properly taking account of structural uncertainty is a challenge for any PSM [18]. It is hoped that a future version can provide a means of conducting PSA that takes these factors into account, for example, by model averaging [19]. We suggest in the meantime that the analyst reports deterministic results using different parametric functions.

## Results

An example using INES is given based on the Destiny-Breast03 study [20]. This clinical study compared Trastruzumab Deruxtecan (intervention) versus Trastruzumab Emtansine (control) for second-line treatment of advanced HER2 + breast cancer [20]. The clinical study reported Kaplan–Meier curves for PFS and OS up to 34 months, though less than 10% of patients had follow-up beyond 2 years. The reported HR were 0.55 (0.36–0.86) for OS and 0.28 (0.22–0.37) for PFS. A template pre-loaded with the data used in this example can be downloaded from https://data.mendeley.com/datasets/tn84rck94z/1 [21].

The INES tool produces interactive graphs comparing the Kaplan–Meier with the user’s parametric choices (Fig. 2). For illustrative purposes, the Weibull was chosen for PFS and OS, and a 100 month time horizon (This article does not comment on the appropriateness of these parameters in this particular case. The reader should consult methodological guidelines [12]). The graph also shows the AIC and the p-value of the test for proportional hazards.

### Unit costs and resource use

Trastruzumab Emtansine is administered 3.6 mg/kg once every 3 weeks and Trastruzumab Deruxtecan is administered 5.4 mg/kg, until progression. For a 100 mg unit with no wastage, for a representative 70 kg individual this represents 2.52 units per treatment cycle of Trastruzumab Emtansine (control: treatment = 2) versus 3.78 units per cycle of Trastruzumab Deruxtecan (intervention: treatment = 1). The list price of a 100 mg unit is (approximately) 2720€ for Trastruzumab Emtansine and 2194€ for Trastruzumab Deruxtecan. The costs per month were coded in the format shown in Additional file 2 assuming a lower bound for prices of 50% of list price. Values are shown here for illustration only and have no bearing with real prices that might be paid.

The costs of a hypothetical subsequent therapy after progression are captured in the parameter “next_line”. This is valued as zero in the base case, and 3000€ per month as a sensitivity analysis.

### Estimate of mean costs and effects

INES reports predictions of survival (Additional file 3), undiscounted and discounted means of life years, costs and QALY (or LY if utilities are 1) (Additional file 4). In this case, the ICER is 248,409€ per incremental life year.

### Univariate sensitivity analyses

INES produces a tornado chart (Additional file 5). Users can select which variables to include or exclude from the display. All charts and graphs can be downloaded as images from the application to the user’s hard drive.

## Discussion

INES provides a tool for users who are knowledgeable about the disease area, the evidence base and the principles of economic evaluation but do not have the time or programming expertise to construct and validate a de novo model. Existing R commands and packages are available to recreate IPD from the Kaplan–Meier coordinates, fit parametric survival functions to these IPD, and construct a PSM with these survival functions. However, taken together, they demand a high level of technical skill and time. INES integrates all these features in a single application that interacts with the user via their browser, without requiring any knowledge by the user of the underlying R commands. INES is unique in this important regard.

INES was designed to offer powerful functionality, previously accessible only to computer programming specialists, and at the same time to be simple to use. The need to integrate the various modules in a single application meant that certain parameters were fixed deliberately in the construction of INES and so cannot be altered by the user. The following paragraphs discuss these and other limitations.

INES implements a PSM with three states. There are a few examples of PSM with four states [22], but three states is the most common configuration for practical purposes [5]. The cycle length is fixed at one month. This need not be a severe limitation for most applications, as the resource use can be defined at very granular intervals.

PSM have been widely used in health technology assessment, particularly in late stage and metastatic cancer. Their popularity has been attributed their simplicity of structure, their frugal demand for input data, and the ability to directly compare results to the source clinical study [5]. Their weaknesses are also well documented. A PSM is mainly descriptive, that is, it does not incorporate an explanation of how a treatment achieves its results. In principle, a treatment that delays disease progression might be expected to delay death, but (unlike a Markov model) a PSM does not parameterize this conditional probability. Survival and PFS in a PSM are modelled  as if they were independent variables.

Models such as PSM are often desirable to extrapolate survival and estimate costs when clinical trial data do not follow up all participants until death [23]. A PSM is usually appropriate when the extrapolation is over a moderate time horizon beyond that of the source clinical study. Over a much longer time horizon, other variables not captured by the clinical study data will influence outcomes (such as mortality related to old age) [24]. In such situations, a Markov model or discrete event simulation may be needed to model these kinds of events. The AIC is only one possible indicator of the appropriateness of the model, and other criteria should also be taken into account.

INES is only appropriate where hazards are proportional. There will be examples where proportional hazards cannot be assumed e.g. if the survival curves radically diverge or converge [5, 25]. In these circumstances an alternative modelling package should be considered. Guidelines are available elsewhere [10, 12].

In Fig. 2, at 100 months, about 15% of the intervention cohort was predicted to still be alive. Truncated mean survival may underestimate the benefit of treatment. However, in the example shown, the model fails if a longer time horizon is chosen, because the OS probability becomes less than PFS, producing a logical inconsistency which halts the model engine (the screen turns grey when the model engine fails, and INES must be restarted from the run.bat file). This is not a failure of INES as such but an inherent limitation of the PSM framework, by modelling OS and PFS as if they were independent. The literature contains ad-hoc attempts to “correct” the problem, for example, by constraining PFS to be less than OS, or specifying that gains in PFS are automatically translated into equal gains for OS [5, 25]. However, these corrections are not based on principle [25] and we decided that it is more transparent if the model generates an error, rather than give an artificially adjusted result. There may be cases where the model successfully runs for the base case but fails for the univariate sensitivity analyses. If the model fails, the user might choose a shorter time horizon, different parametric survival functions, or a different hazard ratio.

If it is important that the model captures explicitly the relation between PFS and OS, then a different framework would be required, such as a Markov model. Likewise, a PSM supposes that the outcomes (progression and mortality) observed in the source clinical study would be replicated in real-world clinical practice. A PSM is not easily adapted to take account of potential biases in the clinical study, such as unrepresentative selection of patients, inappropriate treatment switching, off-label downstream treatments post-progression, drug wastage, or inappropriate comparator treatments [5].

Because a PSM models PFS and OS independently, an increase in PFS can increase costs (where therapies are given until progression) but does not increase OS in the model (e.g. see the tornado chart, Additional file 5). This is another limitation of the PSM framework, rather than INES per se. The tornado shows that a variation in the HR for OS appears “unbalanced”, in the sense that a given increase in the HR increases the ICER much more than the same unit reduction in the HR reduces the ICER. This occurs because as the HR approaches 1, the absolute difference in life years approaches zero, and hence the ICER approaches infinity. This illustrates a limitation of using the ICER (a ratio) as an outcome variable. Furthermore, if the HR becomes greater than 1, the ICER would become negative, which has no interpretation for decision making. Hence users need to take account of the absolute incremental cost and incremental QALY as well as the ICER when interpreting the results of univariate sensitivity analyses.

There are many ways that a PSM could be configured [5, 12, 18, 19, 25]. We aimed to create a tool for survival analysis and PSM that was easy to use and robust. INES can provide a rapid and transparent analysis that can be shared by all parties, for example facilitating the negotiation of price and reimbursement contracts [26]. It is hoped that the tool will be acceptable in many evaluations, but it will not be suitable for all, and potential users should be aware of its limitations (see Table 3). Further work might include time-varying utilities, budget impact assessment, PSA, and model averaging. As the tool is open-source, it is hoped that a community of users will emerge to comment and modify.

## Supplementary Information

**Additional file 1.** Data entry sheet for the price and posology of daratumumab in the ANDROMEDA trial in the progression-free state for a maximum of 24 treatment cycles of 4 weeks each.

**Additional file 2.** Resource use and unit costs.

**Additional file 3. **Model predictions of Progression Free Survival, Post-Progression Survival and deaths.

**Additional file 4.** Undiscounted and discounted results of the model over a time horizon of 100 months.

**Additional file 5.** Tornado chart showing the effect of changes in parameters on the incremental cost-effectiveness ratio.

## Data Availability

All materials are publically available. The application and code are downloadable from https://freeinesapp.github.io. A tutorial dataset is available at Epstein, David (2023), “Tutorial dataset for INES”, Mendeley Data, V1, https://doi.org/10.17632/tn84rck94z.1. A manual is available in Spanish from  https://hdl.handle.net/10481/82594. INES is available freely for commercial and non-commercial use under a creative  commons license. The authors are not responsible for any losses arising from the use of this software.
