# Supplementary material for: INES: Interactive tool for construction and extrapolation of partitioned survival models
Source: Cost Eff Resour Alloc. 2023 Jul 31;21:48. doi: 10.1186/s12962-023-00456-6 (PMC10391963; doi:10.1186/s12962-023-00456-6)
Supplement: Supplementary file 1 — Additional file 1. Data entry sheet for the price and posology of daratumumab in the ANDROMEDA trial in the progression-free state for a maximum of 24 treatment cycles of 4 weeks each. [file 12962_2023_456_MOESM1_ESM.docx]

Additional file 1. Data entry sheet for the price and posology of daratumumab in the ANDROMEDA trial in the progression-free state for a maximum of 24 treatment cycles of 4 weeks each

| parameter | Value (b) | Low | High | Treat-ment (a) | state | unit_ cost | calendar | from | to | by |
| --- | --- | --- | --- | --- | --- | --- | --- | --- | --- | --- |
| Daratumumab | 1500 | 500 | 2000 | 1 | PROGRESSION_ FREE_ SURVIVAL | 1 | weeks | 1 | 8 | 1 |
| Daratumumab | 1500 | 500 | 2000 | 1 | PROGRESSION_ FREE_ SURVIVAL | 1 | weeks | 10 | 24 | 2 |
| Daratumumab | 1500 | 500 | 2000 | 1 | PROGRESSION_ FREE_ SURVIVAL | 1 | weeks | 28 | 96 | 4 |
| Next_line | 1000 | 0 | 2000 |  | POST_TREATMENT_SURVIVAL | 1 | months |  |  |  |

1. if treatment is left blank then this is understood to apply to both arms
2. Note: prices shown here bear no relation to real prices

Explanatory notes for completing the resource use and unit cost tables in INES shown in Supplementary Table 4

- “parameter” refers to the name of the resource, in this case, the drug. This should start with a letter, not a number or other character. For example 5-FU would not be valid input
- “value” refers to the price, along with the “low” and “high” range for univariate sensitivity analysis. Although the quantity consumed of the parameter can change over time, the unit price must be constant
- “treatment” refers to the treatment group, which must be coded 1 for the intervention group or 2 for the control group
- “state” refers to the model state, which must be coded only using the terms “PROGRESSION_FREE_SURVIVAL” or “POST_TREATMENT_SURVIVAL” (note the underscores)
- “unit_cost” refers to the number of units administered per time period (days, weeks or months as appropriate). Note that this is a physical quantity, not a monetary value
- “calendar” specifies the measurement of time over which the quantity referred to in unit_cost is consumed. It can be coded “days”, “weeks” or “months”
- “from” and “to” refer to “calendar” time periods since the start of the model when the quantities are consumed
- “by” refers to the frequency of administration, expressed in “calendar” time periods.

Hence the combination of data in the first row of Supplementary Table 4 (from=1, to=8, by=1) means 1 unit is administered in weeks 1,2,3,4,5,6,7 and 8 (the 1^st^ and 2^nd^ treatment cycles). The data in the second row of Supplementary Table 4 (from=10, to=24, by=2) means 1 unit is administered in week 10,12,14,16,18,20,22 and 24 (the 3^rd^ to the 6^th^ treatment cycle). The combination in the third row of Table 4 (from=28, to=96, by=4) means 1 unit is administered in week 28,32,36 etc. up to week 96 (the 7^th^ to the 24^th^ treatment cycle)

The data input template assumes that the resource is administered on the first day of the specified period. If the patient ceases to be in the PFS state in the model, the consumption of all resources associated with that state automatically discontinues. In the example given, daratumumab is given for a maximum of 96 weeks. In cases where the drug is administered until progression with no other time limit, then the final cell of the series in the “to” field should be left blank. The calendar variable must be the same in each line for a given parameter in a given state (but different resources can have different “calendars” of administration). Consumption can vary over time in the PFS state, as shown in Supplementary Table 4.

After progression (“POST_TREATMENT_SURVIVAL”), the frequency of use of each resource must be constant. Hence the “from” and “to” fields should be left blank (although the “by” field could be used). Like Markov models, PSM do not record when progression occurs, and so cannot keep track of time in the post progression state.
