# Supplementary material for: INES: Interactive tool for construction and extrapolation of partitioned survival models
Source: Cost Eff Resour Alloc. 2023 Jul 31;21:48. doi: 10.1186/s12962-023-00456-6 (PMC10391963; doi:10.1186/s12962-023-00456-6)
Supplement: Supplementary file 2 — Additional file 2. Resource use and unit costs. [file 12962_2023_456_MOESM2_ESM.docx]

Additional file 2. Resource use and unit costs

| *parameter* | *value* | *low* | *high* | *treat-ment* | *state* | *unit_ cost* | *calendar* | *from* | *to* | *by* |
| --- | --- | --- | --- | --- | --- | --- | --- | --- | --- | --- |
| Emtansine | 2720 | 1360 | 2720 | 2 | PROGRESSION_FREE _SURVIVAL | 2.52 | weeks | 1 |  | 3 |
| Deruxtecan | 2194 | 1097 | 2194 | 1 | PROGRESSION_FREE _SURVIVAL | 3.78 | weeks | 1 |  | 3 |
| next_line | 0 | 0 | 3000 |  | POST_PROGRESSION_  SURVIVAL | 1 | months |  |  |  |

Note: prices shown here bear no relation to real prices
