# Supplementary material for: INES: Interactive tool for construction and extrapolation of partitioned survival models
Source: Cost Eff Resour Alloc. 2023 Jul 31;21:48. doi: 10.1186/s12962-023-00456-6 (PMC10391963; doi:10.1186/s12962-023-00456-6)
Supplement: Supplementary file 3 — Additional file 3. Model predictions of Progression Free Survival, Post-Progression Survival and deaths. [file 12962_2023_456_MOESM3_ESM.docx]

Additional file 3: Model predictions of Progression Free Survival, Post-Progression Survival and deaths


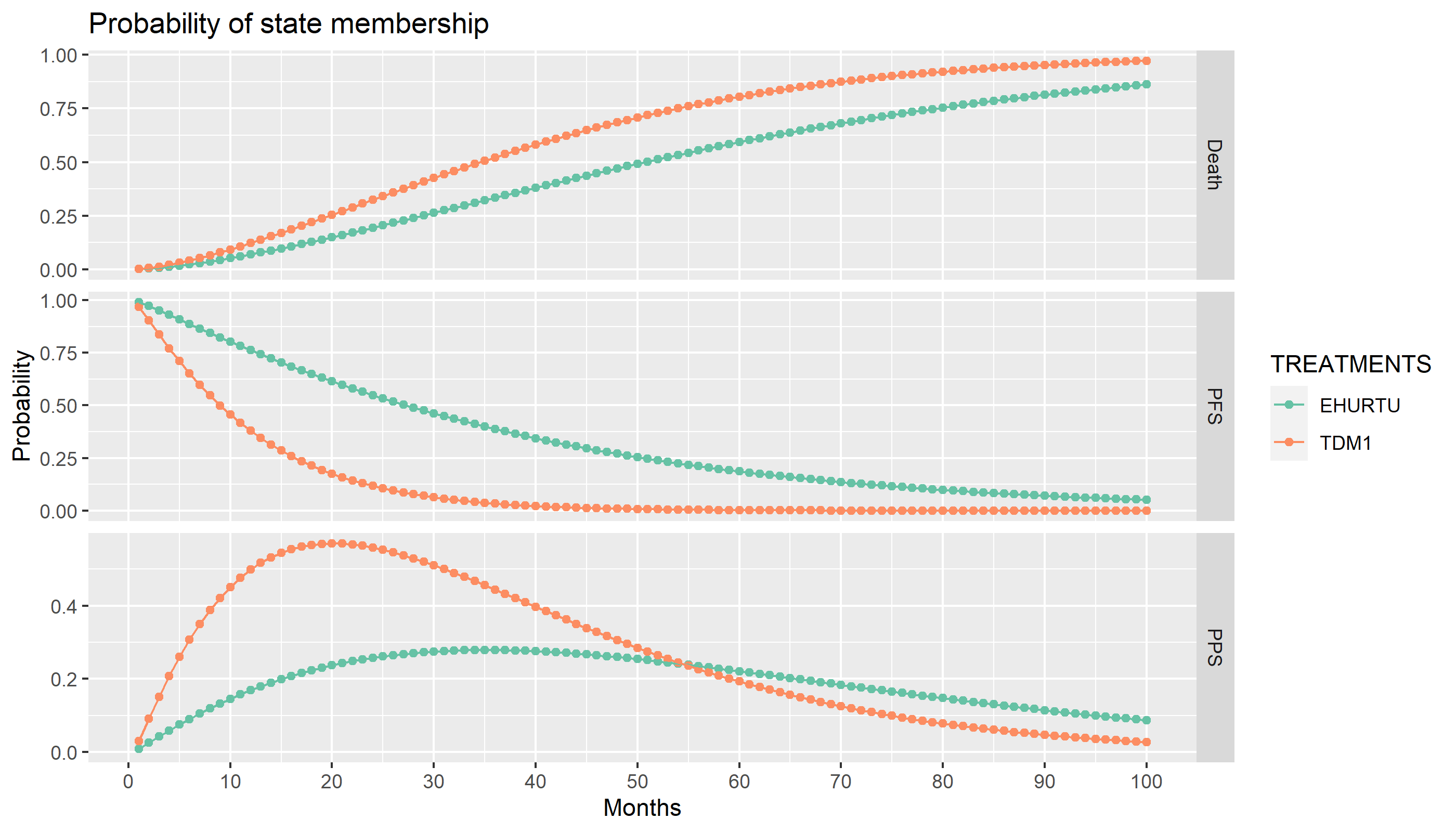


PFS Progression free survival. PPS Post progression survival
