# Supplementary material for: INES: Interactive tool for construction and extrapolation of partitioned survival models
Source: Cost Eff Resour Alloc. 2023 Jul 31;21:48. doi: 10.1186/s12962-023-00456-6 (PMC10391963; doi:10.1186/s12962-023-00456-6)
Supplement: Supplementary file 4 — Additional file 4. Undiscounted and discounted results of the model over a time horizon of 100 months. [file 12962_2023_456_MOESM4_ESM.docx]

Additional file 4: Undiscounted and discounted results of the model over a time horizon of 100 months

Undiscounted Results, Years

Progression Free Survival PFS

Post Progression Survival PPS

Overall Survival OS

PFS TT1: 2.89 PFS TT2: 0.954 Difference: 1.936

PPS TT1: 1.719 PPS TT2: 2.369 Difference: -0.65

OS TT1: 4.609 OS TT2: 3.323 Difference: 1.286

Discounted Results, Years

Cost TT1: 390776 Cost TT2: 112267 Incremental cost: 278510

Effect TT1: 4.229 Effect TT2: 3.108 Incremental QALY: 1.121

ICER: 248409
