# Supplementary material for: INES: Interactive tool for construction and extrapolation of partitioned survival models
Source: Cost Eff Resour Alloc. 2023 Jul 31;21:48. doi: 10.1186/s12962-023-00456-6 (PMC10391963; doi:10.1186/s12962-023-00456-6)

Additional file 5. Tornado chart showing the effect of changes in parameters on the incremental cost-effectiveness ratio


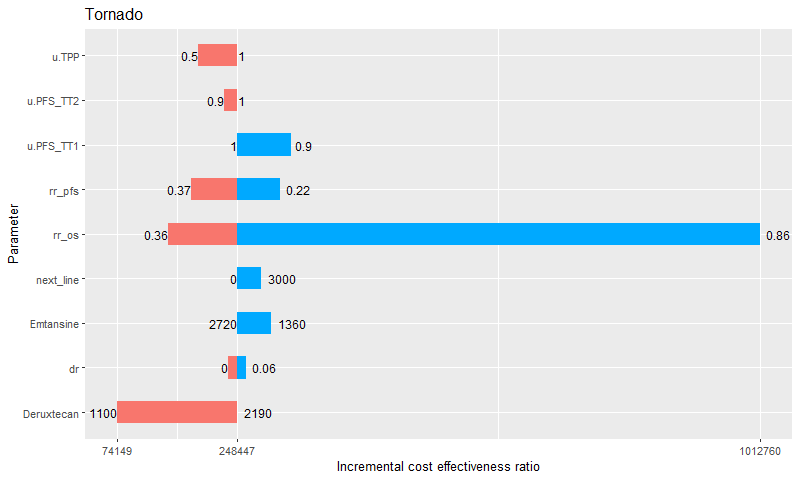

Supplement: Supplementary file 5 — Additional file 5. Tornado chart showing the effect of changes in parameters on the incremental cost-effectiveness ratio. [file 12962_2023_456_MOESM5_ESM.docx]
